# Supplementary material for: Atomistic mechanism of phase transformation between topologically close-packed complex intermetallics
Source: Nat Commun. 2022 May 5;13:2487. doi: 10.1038/s41467-022-30040-0 (PMC9072387; doi:10.1038/s41467-022-30040-0)
Supplement: Supplementary file 1 — Supplementary Information [file 41467_2022_30040_MOESM1_ESM.pdf]

Supplementary Information

Atomistic Mechanism of Phase Transformation between Topologically Close-packed Complex Intermetallics

Huixin Jin[1,2,3,4,5], Jianxin Zhang[2*], Pan Li[2,6], Youjian Zhang[2,7], Wenyang Zhang[2], Jingyu Qin[2], Lihua Wang[1], Haibo Long[1], Wei Li[1], Ruiwen Shao[5], En Ma[3*], Ze Zhang[1,4] and Xiaodong Han[1*]

[1] Institute of Microstructure and Property of Advanced Materials, Beijing University of Technology, Beijing 100124, China

[2] School of Materials Science & Engineering, Shandong University, Jinan 250061, China

[3] Center for Alloy Innovation and Design (CAID), State Key Laboratory for Mechanical Behavior of Materials, Xi'an Jiaotong University, Xi'an 710049, China

[4] School of Materials Science & Engineering, Zhejiang University, Hangzhou 310058, China

5 Beijing Advanced Innovation Center for Intelligent Robots and Systems and Institute of Engineering Medicine, Beijing Institute of Technology, Beijing 100081, China

[6] Institute of Systems Engineering, AMS, PLA, Beijing, 100000, China

[7] Shandong Laboratory of Yantai Advanced Materials and Green Manufacturing, Yantai 264006, PR China

Correspondence:

xdhan@bjut.edu.cn; jianxin@sdu.edu.cn; maen@xjtu.edu.cn

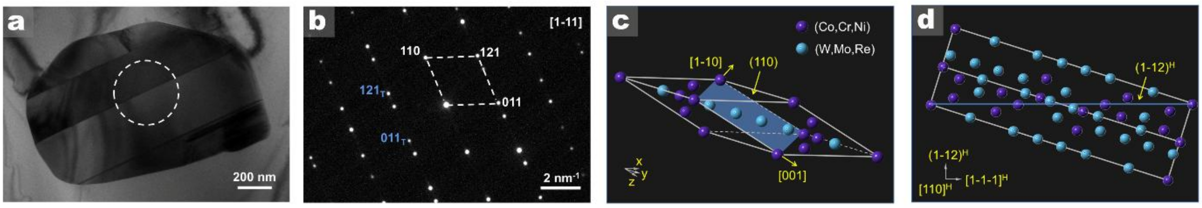

Supplementary Fig. 1 | The μ phase in a Ni-based superalloy. a, The bright-field TEM micrograph of a μ-phase particle. **b,** The selected area electron diffraction (SAED) pattern from the region circled in (**a**). **c,** Atomic structure of rhombohedral unit cell of μ phase. **d,** The atomic packing of the μ phase can also be described using a hexagonal unit cell scheme. The H crystal symmetry shown here is also a common representation of the μ structure, alternative to the rhombohedral unit cell in (**c**). Some typical plane/orientation relationships are: $(1\text{-}12)^{H}$ corresponds to (110) in (**c**), $[110]^{H}$ corresponds to [1-10], and $[1\text{-}1\text{-}1]^{H}$ corresponds to [001].

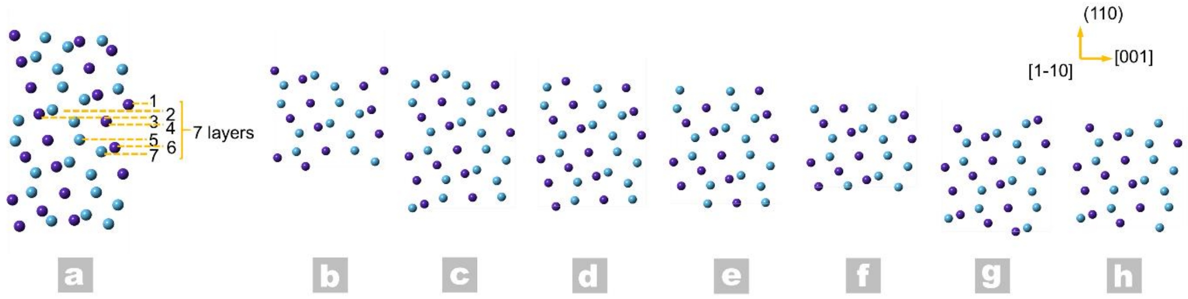

Supplementary Fig. 2 | The atomic structural models of μ-Co_7W_6 (110) slab, viewed along [1-10] projection. The purple and blue atoms represent Co and W, respectively. **a,** The perfect structure of the μ phase. There are 7 different surface configurations, marked with 7 orange dashed lines in (**a**). (**b-h**) are configurations for Type 1~7 surfaces, corresponding to the ones indicated by orange dotted line 1 through 7 in (**a**). Note that the hexagonal description is also often used for the rhombohedral μ phase. Some typical plane/orientation relationships are: (110) corresponds to $(1\text{-}12)^H$, [001] corresponds to $[1\text{-}1\text{-}1]^H$, and [1-10] corresponds to $[110]^H$. Please refer to Supplementary Fig. 1 for details.

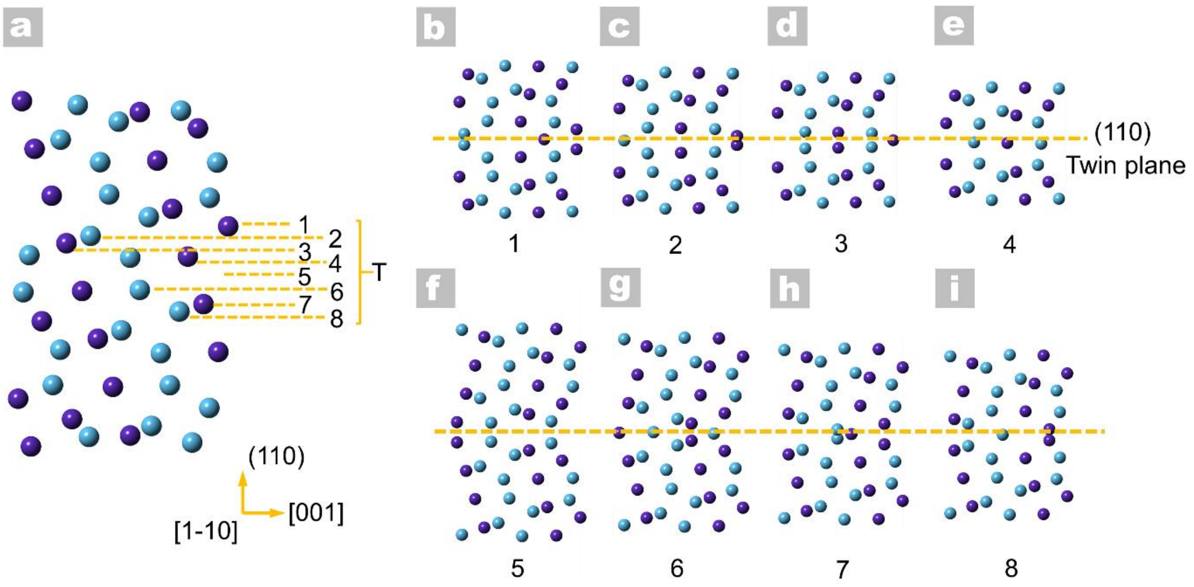

Supplementary Fig. 3 | Schematics of the [1-10] projection of the eight interface structural models with (110) twinning structure. The purple and blue atoms represent Co and W, respectively. **a,** The perfect structure of the μ phase. There are 8 possible different (110) twinning structures, marked with orange dashed lines as the twin boundary. **b-i,** The twinning structures 1 through 8, corresponding to the interface positions indicated by the orange dotted lines 1 to 8 in (**a**). Note that the hexagonal description is also often used for the rhombohedral μ phase. Some typical plane/orientation relationships are: (110) corresponds to $(1\text{-}12)^{H}$, [001] corresponds to $[1\text{-}1\text{-}1]^{H}$, and [1-10] corresponds to $[110]^{H}$. Please refer to Supplementary Fig. 1 for details.

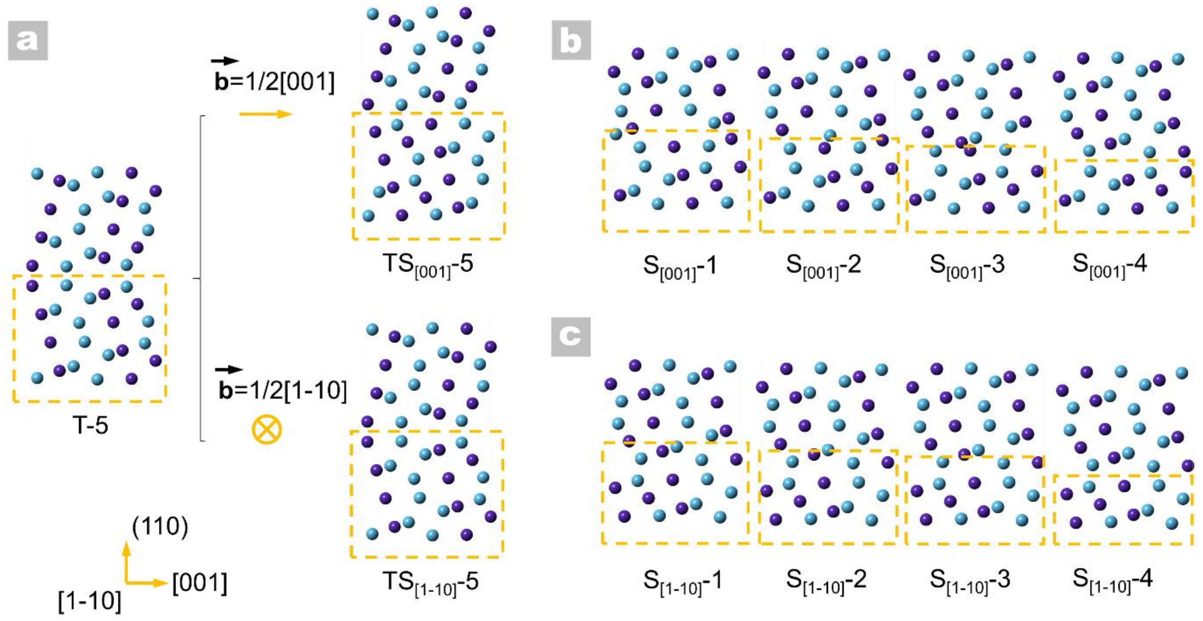

Supplementary Fig. 4 | Schematics of the [1-10] projection of representative interface models with (110) defects. The purple and blue atoms represent Co and W, respectively. a, FTBs formed by twinning (for easy tracking, a block is enclosed with orange dotted box) based on (110) twinning structure sliding along [001] for 1/2[001], or along [1-10] for 1/2[1-10]. Taking twinning structure T-5 as an example, FTBs formed by twinning block's sliding are marked as $TS_{[001]}$-5 and $TS_{[1\text{-}10]}$-5. **b,** The typical stacking fault structural models formed by the block of μ phase (enclosed with orange dotted box) sliding along [001] for 1/2[001]. **c,** The typical stacking fault structural models formed by the block of μ phase sliding along [1-10] for 1/2[1-10]. Note that the hexagonal description is also often used for the rhombohedral μ phase. Some typical plane/orientation relationships are: (110) corresponds to $(1\text{-}12)^H$, [001] corresponds to $[1\text{-}1\text{-}1]^H$, and [1-10] corresponds to $[110]^H$. Please refer to Supplementary Fig. 1 for details.

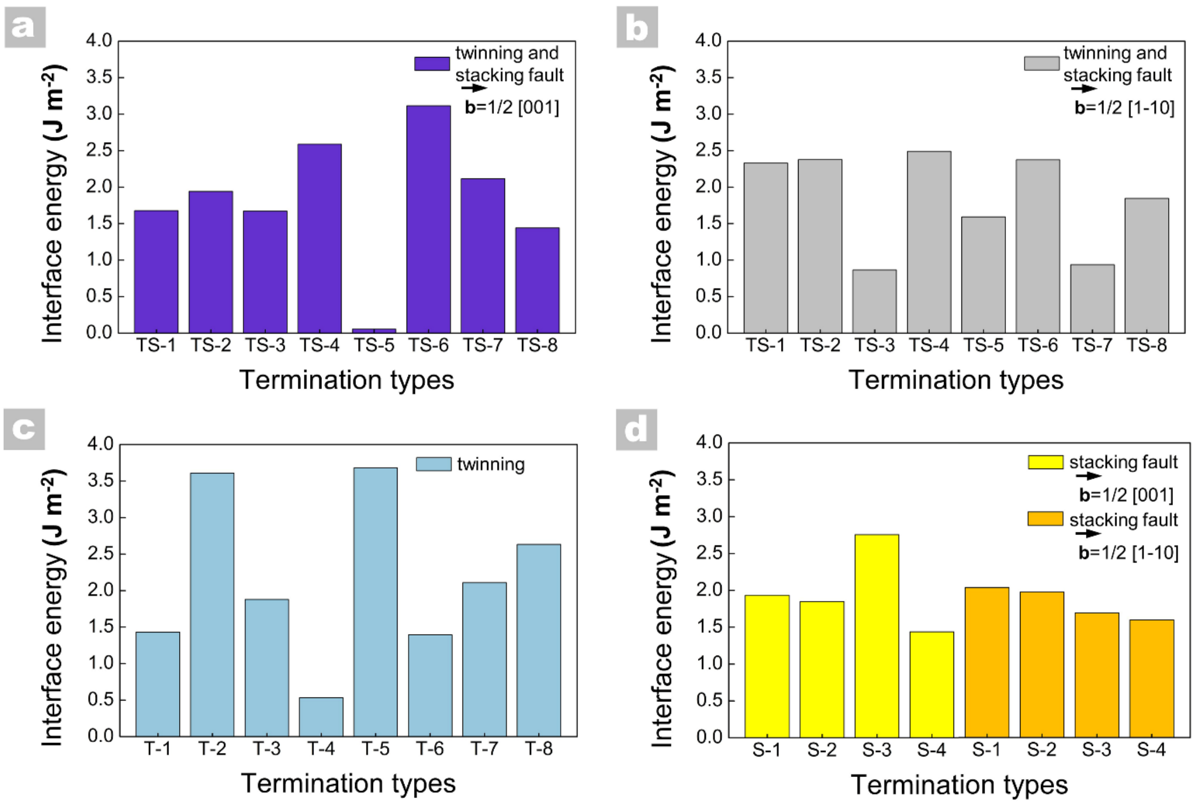

Supplementary Fig. 5 | Interfacial energy of 32 models with (110) defects. a, Interfacial energy of 8 interface models formed by sliding along [001] for 1/2[001] based on the (110) twinned structures. **b,** Interfacial energy of 8 interface models formed by sliding along [1-10] for 1/2[1-10] based on (110) twinned structures. **c,** Interfacial energy of 8 twinned structures. **d,** Interfacial energy of 8 single stacking fault structures; 4 of them are formed by sliding along [001] and 4 of them by sliding along [1-10].

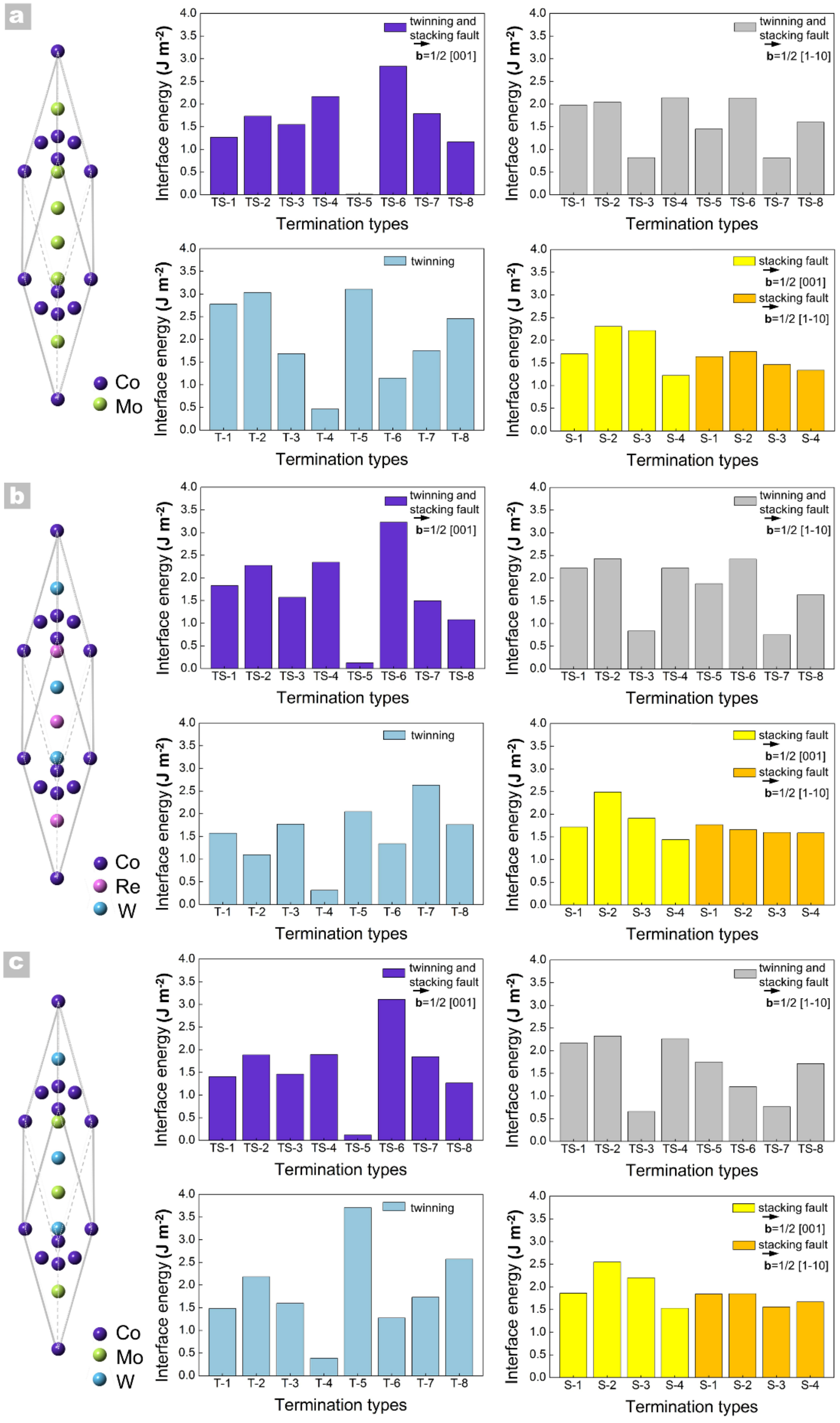
a
b
c
Co
Mo
Re
W
Interface energy (J m-2)
Termination types
twinning and stacking fault b=1/2 [001]
twinning and stacking fault b=1/2 [1-10]
twinning
stacking fault b=1/2 [001]
stacking fault b=1/2 [1-10]
TS-1 TS-2 TS-3 TS-4 TS-5 TS-6 TS-7 TS-8
T-1 T-2 T-3 T-4 T-5 T-6 T-7 T-8
S-1 S-2 S-3 S-4 S-1 S-2 S-3 S-4

Supplementary Fig. 6 | Interfacial energy of (110) defects in the μ phase models with three constituent components (Co_7Mo_6, $Co_7W_3Re_3$, and $Co_7W_3Mo_3$). The 32 interface structures of (110) defects of each type of μ phase in (**a-c**) are similar to those in Supplementary Fig. 5.

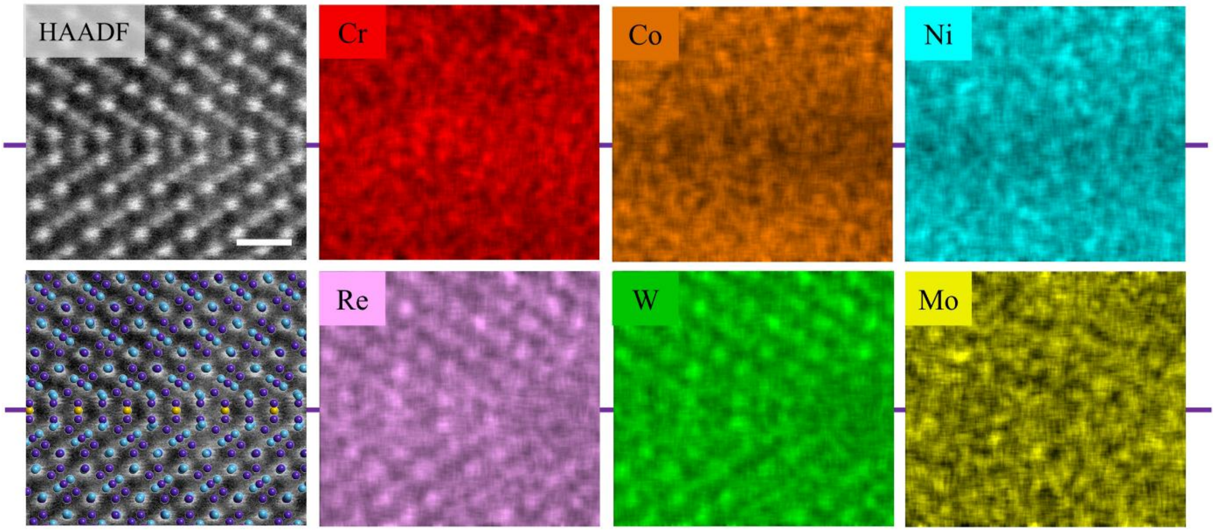

Supplementary Fig. 7 | Chemical mapping indicating the element distribution of the product (partial structure of P phase) after the structural transformation. The lower left corner panel is the atomic schematic of the structure. Note the location of the FTB (purple line). The yellow atoms are the extra row of atoms that have diffused in, and the 3d metal atoms dominated by Cr correspond well with these positions. Scale bar is 0.5 nm.

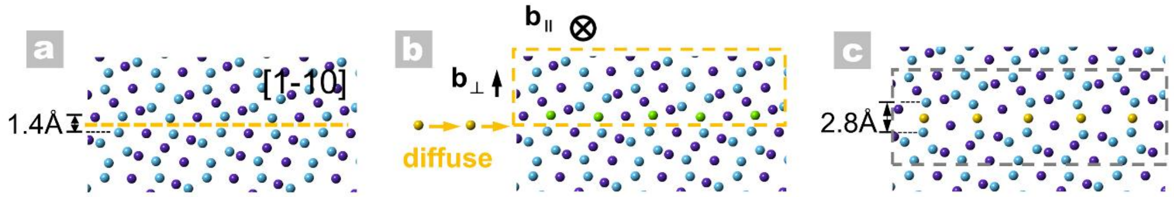

Supplementary Fig. 8 | Projections of atomic arrangement of the crystal structures in Figure 2e-g along the [1-10]$_{\mu}$ zone axis of the μ phase. a, Atomic arrangement at the FTB of the μ phase along [1-10]$_{\mu}$. Note that the hexagonal description is also often used for the rhombohedral μ phase. Some typical plane/orientation relationships are: (110) corresponds to (1-12)^{H}, and [1-10] corresponds to [110]^{H}. Please refer to Supplementary Fig. 1 for details. The orange dotted line marks the interface of the FTB, where the additional incoming atoms are inserted. **b,** Going from (**a**) to (**c**), upon the extra atoms gradually diffusing in, taking all the "interstitial"-like positions (with the same vertical coordinates underneath the green atoms) on the plane pointed by the arrows, the atoms inside the entire orange dotted box would have been displaced horizontally into the plane of the paper by Burgers vector $\mathbf{b}_{//}$ and upwards by $\mathbf{b}_{\perp}$, transforming the structure from (**a**) to (**c**) with minor relaxation. Here the green atoms in (**b**) are the blue atoms inside the atomic columns marked with red dashed circles (there are also purple atoms in addition to the blue ones) in Fig. 2f. **c,** The resultant atomic arrangement after these actions, showing that the atoms inside the grey dotted box have reconfigured into the partial structure of P (or σ) phase.

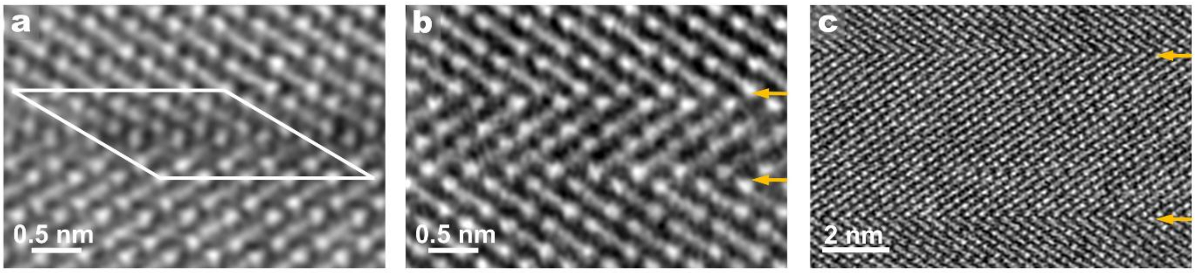

Supplementary Fig. 9 | Closed Burgers circuit around the (110) FTB of the μ phase and the examples of the distribution of FTBs with different spacings. The white closed Burgers circuit in (**a**) serves as the reference, with respect to which the orange Burgers circuit (with closure failure) in Fig. 3 was drawn. **b,** Two FTBs with a relatively small spacing (i.e., 2D). **c,** Two FTBs with a relatively large spacing. The orange arrows mark the interface position of the FTBs in (**b-c**).

Supplementary Table 1 | Surface energy of Co_7W_6 (110) surfaces as a function of atomic layers

Termination	Atomic layers (N) and surface energy (J m^{-2})		
Type 1	8	15	22
	3.13	3.17	3.16
Type 2	13	20	27
	3.32	3.30	3.30
Type 3	11	18	25
	3.24	3.26	3.26
Type 4	9	16	23
	3.28	3.29	3.30
Type 5	7	14	21
	3.06	3.10	3.11
Type 6	12	19	26
	3.27	3.27	3.26
Type 7	10	17	24
	3.32	3.36	3.36

Supplementary Table 2 | The crystallographic information[1-4] for the μ, P and σ phases and their orientation relationships[5,6]

Type	Crystallographic information				Lattice correspondence
μ-R	Space group		R-3m (no. 166)		$(110)_{\mu}$//$(1\text{-}12)^{H}$//$(100)_{P}$//$(100)_{\sigma}$
	Crystal structure		Rhombohedral		$[1\text{-}11]_{\mu}$//$[42\text{-}1]^{H}$//$[011]_{P}$//$[011]_{\sigma}$
	Lattice parameters		a=b=c=8.94Å		$[001]_{\mu}$//$[1\text{-}1\text{-}1]^{H}$//$[001]_{P}$//$[010]_{\sigma}$
			α=β=γ=30.7°		$[1\text{-}10]_{\mu}$//$[110]^{H}$//$[010]_{P}$//$[001]_{\sigma}$
	This work		a=b=c=8.94Å		
			α=β=γ=30.7°		
	Atoms per unit cell		13		
	Wyckoff position	x	y	z	
	1a	0	0	0	
	$2c_1$	0.167	0.167	0.167	
	$2c_2$	0.346	0.346	0.346	
	$2c_3$	0.448	0.448	0.448	
	6h	0.090	0.090	0.590	
μ-H	Space group		R-3m (no. 166)		
	Crystal structure		Hexagonal		
	Lattice parameters		a=b=4.73Å, c=25.54Å		
			α=β=90°, γ=120°		
	Atoms per unit cell		39		
	Wyckoff position	x	y	z	
	3a	0	0	0	
	$6c_1$	0	0	0.167	
	$6c_2$	0	0	0.346	
	$6c_3$	0	0	0.448	
	18h	0.833	0.167	0.257	

P	Space group	Pnma (no. 62)		
	Crystal structure	Orthorhombic		
	Lattice parameters	a=16.90Å, b=4.71Å, c=9.04Å		
		α=β=γ=90°		
	This work	a=16.94Å, b=4.74Å, c=9.02Å		
		α=β=γ=90°		
	Atoms per unit cell	56		
	Wyckoff position	x	y	z
	$4c_1$	0.116	0.250	0.068
	$4c_2$	0.031	0.250	0.517
	$4c_3$	0.251	0.250	0.143
	$4c_4$	0.158	0.250	0.333
	$4c_5$	0.186	0.250	0.598
	$4c_6$	0.346	0.250	0.669
	$4c_7$	0.450	0.250	0.459
	$4c_8$	0.408	0.250	0.194
	$4c_9$	0.080	0.250	0.811
	$4c_{10}$	0.367	0.250	0.941
	$8d_1$	0.288	0	0.383
	$8d_2$	0.536	0.999	0.250
σ	Space group	$P4_2/mnm$ (no. 136)		
	Crystal structure	Tetragonal		
	Lattice parameters	a=b=9.12Å, c=4.72Å		
		α=β=γ=90°		
	Atoms per unit cell	30		
	Wyckoff position	x	y	z
	2a	0	0	0

4f	0.399	0.399	0
$8i_1$	0.464	0.131	0
$8i_2$	0.741	0.066	0
8j	0.183	0.183	0.251

Supplementary Table 3 | Work of separation of 32 possible interface structures of (110) defects (refer to Supplementary Fig. 1 for the hexagonal description of the related crystallographic planes and directions)

Termination	Work of separation (J m^{-2})				
	$TS_{[001]}$	$TS_{[1-10]}$	T	$S_{[001]}$	$S_{[1-10]}$
1	4.81	4.15	5.05	4.55	4.45
2	4.61	4.18	2.95	4.75	4.63
3	4.84	5.65	4.63	3.79	4.86
4	3.78	3.88	5.84	4.76	4.60
5	6.14	5.70	2.52		
6	3.25	4.68	4.98		
7	4.52	4.60	4.53		
8	5.09	3.99	3.90		

Supplementary References:

1 Rae, C. M. F. & Reed, R. C. The precipitation of topologically close-packed phases in rhenium-containing superalloys. *Acta Mater.* **49**, 4113-4125, doi:10.1016/s1359-6454(01)00265-8 (2001).

2 Brink, C. & Shoemaker, D. P. A Variation on the sigma-phase structure - the crystal structure of the p-phase, mo-ni-cr. *Acta Crystallographica* **8**, 734-735, doi:10.1107/s0365110x55002259 (1955).

3 Shoemaker, D. P., Shoemaker, C. B. & Wilson, F. C. The crystal structure of the p phase, mo-ni-cr .2. refinement of parameters and discussion of atomic coordination. *Acta Crystallographica* **10**, 1-14, doi:10.1107/s0365110x57000018 (1957).

4 Joubert, J. M. Crystal chemistry and Calphad modeling of the sigma phase. *Progress In Materials Science* **53**, 528-583, doi:10.1016/j.pmatsci.2007.04.001 (2008).

5 Zhou, D. S., Ye, H. Q. & Kuo, K. H. An hrem study of the intergrowth structures of sigma-related phases and the mu-phase. *Philos. Mag. A-Phys. Condens. Matter Struct. Defect Mech. Prop.* **57**, 907-922, doi:10.1080/01418618808204525 (1988).

6 Tan, X. P. *et al.* Intergrowth of P phase with mu phase in a Ru-containing single-crystal Ni-based superalloy. *Philos. Mag. Lett.* **92**, 556-562, doi:10.1080/09500839.2012.700409 (2012).
