# Supplementary material for: Atomistic mechanism of phase transformation between topologically close-packed complex intermetallics
Source: Nat Commun. 2022 May 5;13:2487. doi: 10.1038/s41467-022-30040-0 (PMC9072387; doi:10.1038/s41467-022-30040-0)
Supplement: Supplementary file 3 — Description of Additional Supplementary Files [file 41467_2022_30040_MOESM3_ESM.pdf]

Description of Additional Supplementary Files

File Name: Supplementary Data 1
Description: Crystal structure of rhombohedral μ-Co_7W_6 phase.

File Name: Supplementary Data 2
Description: Crystal structure of hexagonal μ-Co_7W_6 phase.

File Name: Supplementary Data 3
Description: Crystal structure of σ phase. Since the phase has no fixed element occupation as a reference and its Wyckoff positions is the average of the Wyckoff positions of the σ phases with various components, the elements here are all represented by Ni.

File Name: Supplementary Data 4
Description: Crystal structure of P phase. As with the reason for σ phase, the elements here are all represented by Ni.

File Name: Supplementary Data 5
Description: Crystal structure of (110) FTB observed along $[1\text{-}10]_{\mu}$, corresponding to Fig. 1c. There are vacuum layers on both sides of the structure.

File Name: Supplementary Data 6
Description: Crystal structure of FTBs with a spacing of D, observed along $[1\text{-}10]_{\mu}$. Fig. 4d is the atomic arrangement of this structure projected along $[1\text{-}11]_{\mu}$.

File Name: Supplementary Data 7
Description: Crystal structure of Supplementary Data 6 after transformation. Fig. 4e is the atomic arrangement of this structure projected along $[1\text{-}11]_{\mu}$. In order to facilitate the readers to track the results, the extra atoms that have diffused in are all represented by Cr.

File Name: Supplementary Data 8
Description: Crystal structure of FTBs with a spacing of 2D, observed along $[1\text{-}10]_{\mu}$. Fig. 4g is the atomic arrangement of this structure projected along $[1\text{-}11]_{\mu}$.

File Name: Supplementary Data 9
Description: Crystal structure of Supplementary Data 8 after transformation. Fig. 4h is the atomic arrangement of this structure projected along $[1\text{-}11]_{\mu}$. In order to facilitate the readers to track the results, the extra atoms that have diffused in are all represented by Cr.
